# One-year outcomes of wide antral cryoballoon ablation guided by high-density mapping vs. conventional cryoballoon ablation for atrial fibrillation: a propensity score–matched study

**DOI:** 10.3389/fcvm.2024.1327639

**Published:** 2024-02-01

**Authors:** Sanbao Chen, Zulu Wang, Fengqi Xuan, Ming Liang, Zhiqing Jin, Jian Ding, Mingyu Sun, Ping Zhang, Yaling Han

**Affiliations:** ^1^ Department of Cardiology, The General Hospital of Northern Theater Command, Shenyang, China; ^2^ Department of Cardiology, Beifang Hospital of China Medical University, Shenyang, China

**Keywords:** atrial fibrillation, high-density mapping, pulmonary vein isolation, cryoballoon ablation, carina region

## Abstract

**Background:**

Pulmonary vein isolation with wide antral ablation leads to better clinical outcomes for the treatment of atrial fibrillation, but the isolation lesion is invisible in conventional cryoballoon ablation. In this study, we aim to investigate the efficacy of the wide pulmonary vein isolation technique that includes the intervenous carina region, guided by high-density mapping, compared with pulmonary vein isolation alone without the mapping system.

**Methods:**

We conducted a propensity score–matched comparison of 74 patients who underwent a wide cryoballoon ablation guided by high-density mapping (mapping group) and 74 controls who underwent conventional cryoballoon ablation in the same period (no-mapping group). The primary outcome was a clinical recurrence of documented atrial arrhythmias for >30 s during the 1-year follow-up.

**Results:**

Of 74 patients in the mapping group, residual local potential in the pulmonary vein antrum was found in 30 (40.5%) patients, and additional cryothermal applications were performed to achieve a wide pulmonary vein isolation. Compared with the no-mapping group, the use of the mapping system in the mapping group was associated with a longer fluoroscopic time (26.97 ± 8.07 min vs. 23.76 ± 8.36 min, *P* = 0.023) and greater fluoroscopic exposure [339 (IQR181–586) mGy vs. 224 (IQR133–409) mGy, *P* = 0.012]. However, no significant differences between the two groups were found in terms of procedural duration and left atrial dwell time (104.10 ± 18.76 min vs. 102.45 ± 21.01 min, *P* = 0.619; 83.52 ± 17.01 min vs. 79.59 ± 17.96 min, *P* = 0.177). The rate of 12-month freedom from clinical atrial arrhythmia recurrence was 85.1% in the mapping group and 70.3% in the no-mapping group (log-rank *P* = 0.029).

**Conclusion:**

Voltage and pulmonary vein potential mapping after cryoballoon pulmonary vein isolation can identify residual potential in the pulmonary vein antrum, and additional cryoablation guided by mapping leads to improved freedom from atrial arrhythmias compared with conventional pulmonary vein isolation without the mapping system.

**Clinical Trial Registration Number:**

ChiCTR2200064383.

## Introduction

Wide circumferential electrical pulmonary vein isolation (PVI) by catheter ablation has emerged as a safe and effective treatment for drug-resistant paroxysmal or persistent atrial fibrillation (AF) (1–4). The use of cryoballoon ablation (CB-A) has proved to be non-inferior to radiofrequency ablation (RFA) in terms of efficacy and safety (5, 6). Although cryoballoon was designed for PVI with a single shot, satisfactory freezing for some structures, especially for the intervenous carina region, remains problematic because of a high degree of pulmonary vein (PV) anatomic variability (7–9).

Three-dimensional (3D) mapping systems are usually used to facilitate ablation procedures by reconstructing the atrial anatomy in point-by-point RFA. Theoretically, high-density voltage maps generated by a 3D mapping system after PVI may contribute to the identification of low-voltage signals responsible for AF recurrence and further enhance the success rates of the index procedure (9, 10). However, data for the mapping system in CB-A are limited and sometimes controversial in nature. Some have argued that voltage mapping after standard confirmation of PVI yields no reduction in AF recurrence (11, 12), while others have demonstrated that the detection of incomplete ablation yields promising results (9, 13).

The purpose of this study was to investigate whether wide antral PVI including the carina region under the guidance of a high-density mapping system would improve clinical outcomes of AF compared with PVI without mapping.

## Materials and methods

### Study population

This study was a single-center, propensity score–matched study. Between July 2021 and March 2022, all consecutive patients undergoing CB-A under the guidance of a high-density mapping system (mapping group) in the General Hospital of Northern Theater Command were enrolled. Patients who were eligible but underwent conventional CB-A (no-mapping group) in the same period were chosen as real-world controls, and they were propensity-score-matched to minimize selection bias with patients in the mapping group. The same surgeons with comparable experience performed the procedures in roughly equal proportions.

Patients with drug-resistant AF who underwent a first-time PVI procedure with CB-A were included in the study. The definitions of paroxysmal, persistent, and long-standing persistent AF were based on recent guidelines (14). The exclusion criteria were as follows: (1) previous AF ablation or cardiac surgery; (2) left atrial diameter (LAD) ≥ 50 mm assessed by echocardiography; (3) uncontrolled heart failure; and (4) significant valvular disease. The study protocol was carried out under the ethical principles established by the Declaration of Helsinki and approved by the local ethics committee of our institution. All patients provided written informed consent for the ablation procedure and enrollment in our study.

### Common pathways of the procedure

In general, transesophageal echocardiography was routinely performed the day before ablation to evaluate the presence of thrombi. A transthoracic echocardiography was obtained within 1 week prior to the procedure to assess the left ventricular ejection fraction (LVEF) and intracavitary dimensions. All patients routinely underwent CT angiography for the assessment of the LA and PV anatomy and the distance between the esophagus and the PVs. An electrophysiological study and the ablation procedure were performed under conscious sedation with fentanyl. One right femoral access (an 8.5-F sheath) was used to perform transseptal puncture. A coronary sinus (CS) catheter and a right phrenic nerve pacing catheter were advanced through the femoral veins. A bolus heparin (100 IU/kg) was administered immediately after the venous access and heparinized saline was additionally infused to maintain an activated clotting time of 300–350 s during the procedure.

### Conventional cryoballoon ablation procedure

After gaining access to the LA, a spinal mapping catheter (Achieve, Medtronic) was advanced to each pulmonary vein ostium to obtain the recording of baseline PV potentials. A 28-mm cryoballoon (CB) catheter (Arctic Front Advance, Medtronic Inc.) was advanced, inflated, and positioned at each PV antrum. The 23-mm CB was not used in any patient. After the confirmation of optimal vessel occlusion, which was considered to be achieved when no backflow to the LA was observed after a contrast medium was injected to the PV by using an x-ray image, cryothermal energy application to allow freezing was commenced. One standard CB application lasted 180 s (if no contraindications) and each PV was ablated at least twice. To obtain a lesion set of a wide-area circumferential antrum, the second cryoablation procedure was optimized by the proximal seal technique (15). For some PV anatomy (a PV with a large ostium, common PV ostium, thick carina between ipsilateral PV, etc.), empirical additional cryoapplication was allowed at the discretion of the surgeons. The cryoenergy was delivered in the sequence of the left superior (LS) PV, left inferior (LI) PV, right superior (RS) PV, and right inferior (RI) PV. To prevent right phrenic nerve palsy (PNP), the right phrenic nerve was stimulated using an electrode positioned in the right subclavian vein when ablating the right PV. A bidirectional block between the PVs and the LA was verified by using the Achieve catheter 20 min after the last application in all patients to avoid recovery of cardiac excitability. If it was felt necessary, pacing from the distal CS and/or superior vena cava (SVC) was performed to distinguish far-field atrial activity from the local PV potential recorded on the mapping catheter. No additional applications were used after PVI.

### High-density mapping system–guided ablation procedure

Following transseptal puncture, a detailed image reconstruction of the LA and PV was obtained by using the KODEX-EPD system (Philips, the Netherlands), which has been previously described in detail (16). Standard CB applications were performed for each PV as was done for the no-mapping group (including the application of the proximal seal technique). After the confirmation of isolation for each PV, detailed bipolar voltage amplitude maps (during sinus rhythm) with the 3D mapping system were generated using the Achieve catheter. At least 500 points per geometry were carefully acquired. If AF persisted after the abovementioned cryoablation, electrical cardioversion was performed to restore the sinus rhythm before mapping. An effort was made to distinguish PV-like potentials from local PV potentials to prevent excessive ablation, and suspected near-field PV potentials were confirmed by pacing from the distal CS and/or SVC (17). The PV-LA junction was defined as the point of maximal transition between the LA wall and the PV wall. If the transition between the PV and the LA was gradual, an evaluation was made to determine the point at which the transition was most dramatic (9). In addition, differences in electrical signals in the PV and LA provided information to determine the PV-LA junction. Scar areas were defined as those with voltage amplitudes of less than 0.1 mV. The criteria for the wide-antral ablation area were as follows: (1) all local signals within the PV-LA junction should be <0.1 mV; (2) the scar area of the superior PV should be continuous to that of the inferior PV and both anterior and posterior carina regions should be scarred. According to the voltage map, patients who did not meet the criteria to achieve the wide-antral ablation area would receive an additional freeze cycle of 120–150 s under the guidance of the KODEX system, and the recommended freezing time for the posterior wall of the LA was shortened to 120 s to prevent esophageal injury. To deliver cryoenergy at a certain region of the LA antrum, the Achieve catheter was advanced distally into the PV to provide sufficient support and stability for the balloon (18). Taking the anterior part of the right carina region as an example, the Achieve catheter was positioned distally into the inferior branch of the superior PV and/or the superior branch of the inferior PV as an anchor. Following advancing the inflated balloon over the Achieve catheter, the balloon was pushed toward the desired region by a clockwise rotation of the sheath. Complete occlusion was not necessary, but adequate balloon-tissue contact at the desired region (non-occlusion technique) was confirmed by fluoroscopy and using a contrast injection before freezing. Such ablations were then reassessed with repeat voltage maps immediately and 20 min after the final application of cryoablation.

### Study follow-up

Follow-ups were scheduled at 3, 6, 9, and 12 months after the index procedure. At each patient visit, medical history was obtained, and a physical examination and 12-lead ECG were performed. A 24-h Holter or 7-day Holter was used at 3 and 12 months, while it was not mandatory at 6 and 9 months. A 7-day Holter was available for the12-month follow-up since January, 2023. In case of symptoms that suggested recurrent arrhythmia, additional ECG or 24-h Holter was performed. All patients received antiarrhythmic drugs (AADs) and anticoagulation for 3 months to prevent early recurrences and to reduce the incidence of thromboembolic events. A proton pump inhibitor was provided for at least 6 weeks. The primary outcome was freedom from clinical recurrence of atrial arrhythmias at 12 months after the index procedure. Clinical recurrence was defined as any documented episode of AF, atrial flutter (AFL), or atrial tachyarrhythmia (AT) lasting greater than 30 s (1). An episode of atrial arrhythmias within the first 3-month blanking period after the index procedure was considered early recurrence and only recurrence after the blanking period was diagnosed as clinical recurrence. After the blanking period, Class I or III AADs were prescribed only in the case of clinical recurrence or when frequent atrial premature beats were documented by ECG or 24-h Holter ECG monitoring.

### Statistical analysis

Continuous variables were expressed as means ± standard deviations or median and first to third interquartile range. Categorical variables were expressed as frequencies and percentages (%). Continuous variables were compared using Student’s *t*-test or the Mann–Whitney *U*-test. Categorical variables were compared using the *χ*^2^ test or Fisher's exact test. To reduce treatment-selection bias between the mapping group and the no-mapping group, propensity scores were calculated using binary logistic regression according to baseline characteristics and potential confounders, including age, sex, type of AF (paroxysmal, persistent, or long-standing persistent), body mass index, hypertension, diabetes mellitus, coronary artery disease, heart failure, history of stroke, drinking, smoking, B-type natriuretic peptide (BNP), LAD, and LVEF. Nearest-neighbor matching without replacement was performed on the basis of propensity scores in a 1:1 ratio with calipers of width equal to 0.2 of the standard deviation of the logit of the propensity score. The recurrence-free survival rate over follow-up was calculated using the Kaplan–Meier method and survival distributions were compared between the treatment groups by means of the log-rank test. To assess the robustness of our findings, a sensitivity analysis of the overall cohort was performed by using the multivariate Cox regression model and the univariate Cox model was used for the propensity-score-matched cohort. All covariates previously used for propensity score estimation were included in the multivariate Cox model. The *P*-values were two-sided, and *P*-values of <0.05 were deemed statistically significant. Analyses were conducted using SPSS version 26 (Armonk, NY: IBM Corp), except for propensity score matching (R version 3.5.0, The R Foundation for Statistical Computing, Vienna, Austria).

## Results

### Baseline characteristics

**Figure 1:**
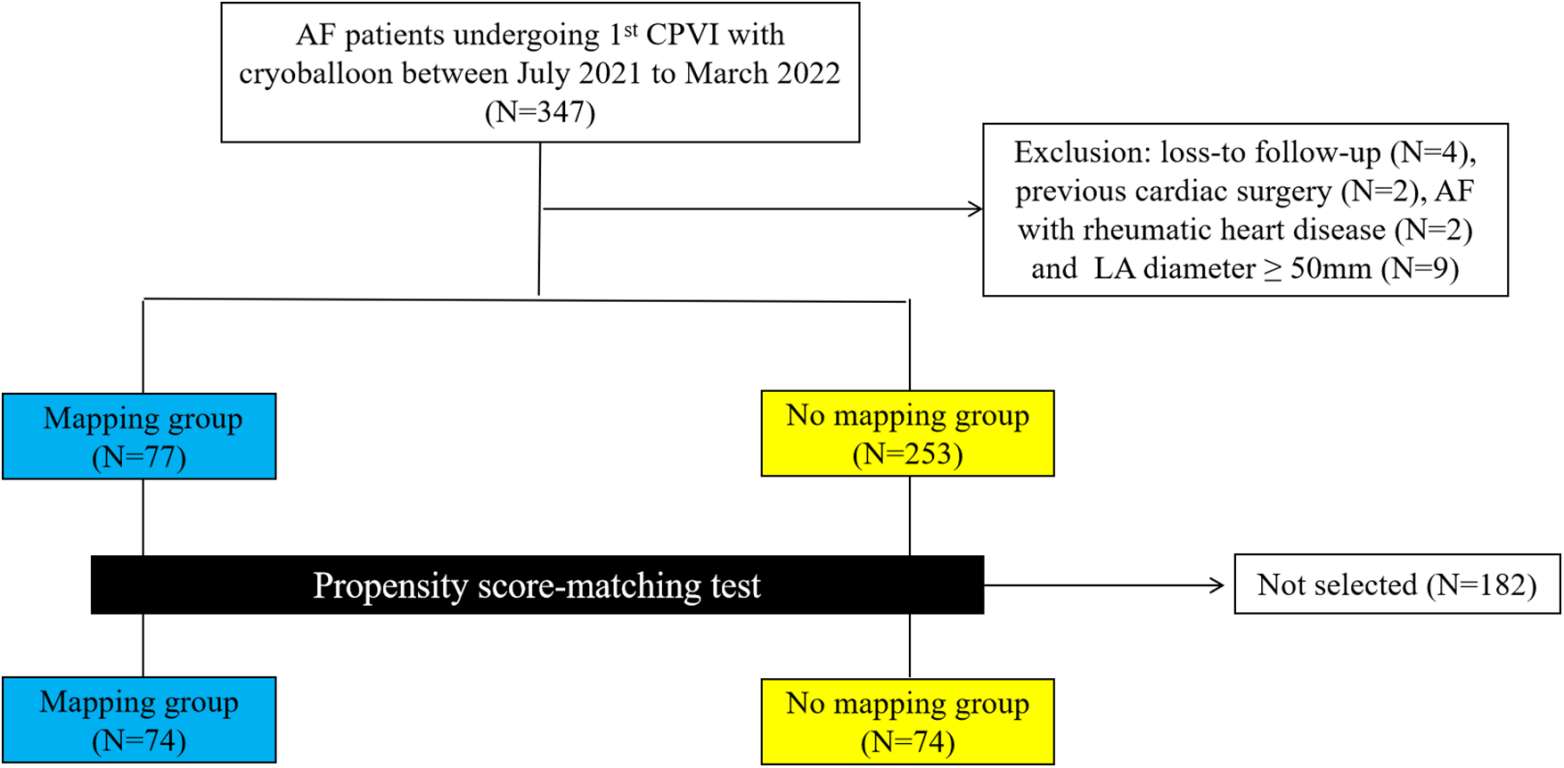
A flowchart of patients enrolled and included in the analyses. AF, atrial fibrillation; CPVI, circumference pulmonary vein isolation; LA, left atrium.

**Figure 2:**
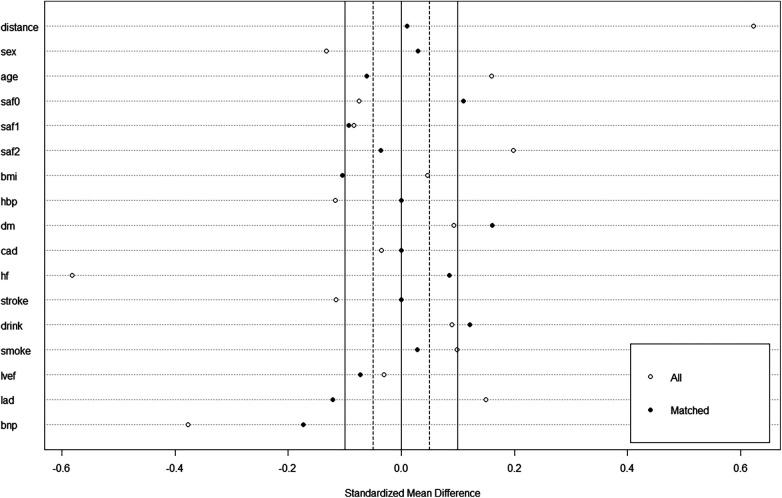
A love plot for expressing standard mean differences for baseline covariates of patients in the mapping group and no-mapping group, before and after propensity score matching.

Among 347 consecutive patients undergoing first–time PVI with CB-A during the period between July 2021 and March 2022, 17 patients were excluded (Figure 1). In the cohort, 74 patients in the mapping group and 74 in the no-mapping group were actually matched in a 1:1 ratio based on propensity scores. All baseline characteristics showed no significant differences between the matched groups (Table 1), and standard mean differences for baseline covariates of patients in the two groups before and after propensity score matching were expressed in a love plot (Figure 2).

**Table 1:** Baseline characteristics before and after matching.

Baseline characteristics	Non-matched group			Matched group		
	Mapping group (*n* = 77)	No-mapping group (*n* = 253)	*P*-value	Mapping group (*n* = 74)	No-mapping group (*n* = 74)	*P*-value
Age, year	60.31 ± 9.71	58.76 ± 10.60	0.253	60.50 ± 9.77	61.09 ± 10.26	0.719
Male	54 (70.1%)	162 (64.0%)	0.324	51 (68.9%)	52 (70.3%)	0.858
BMI, kg/m^2^	25.73 ± 3.44	25.57 ± 3.44	0.762	25.67 ± 3.43	26.03 ± 3.50	0.531
Type of AF						
PAF	44 (57.1%)	154 (60.9%)	0.559	44 (59.5%)	40 (54.1%)	0.507
PerAF	20 (26.0%)	75 (29.6%)	0.533	20 (27.0%)	23 (31.1%)	0.587
Longstanding PerAF	13 (16.9%)	24 (9.5%)	0.072	10 (13.5%)	11 (14.9%)	0.814
HBP	30 (39.0%)	113 (44.7%)	0.337	30 (40.5%)	30 (40.5%)	1
DM	10 (13.0%)	25 (9.9%)	0.438	9 (12.2%)	5 (6.8%)	0.261
CAD	8 (10.4%)	29 (11.5%)	0.794	7 (9.5%)	7 (9.5%)	1
History of stroke	9 (11.8%)	39 (15.4%)	0.417	9 (12.2%)	9 (12.2%)	1
Heart failure	2 (2.6%)	30 (11.9%)	0.016	1 (1.4%)	2 (2.7%)	0.560
Smoking	28 (36.4%)	80 (31.6%)	0.437	25 (33.8%)	24 (32.4%)	0.861
Drinking	21 (27.3%)	59 (23.3%)	0.479	20 (27.0%)	16 (21.6%)	0.443
LVEF, %	59.91 ± 6.91	60.12 ± 7.40	0.825	59.99 ± 7.026	60.49 ± 6.2	0.648
LAD, mm	40.09 ± 5.81	39.22 ± 5.24	0.215	39.70 ± 5.43	40.41 ± 4.62	0.398
NT-pro BNP	150 (131–400)	374 (103–729)	0.022	150 (131–400)	285 (86–726)	0.183

Values are reported as mean ± standard deviation, median, and first to third interquartile range or *n* (%).

BMI, body mass index; AF, atrial fibrillation; PerAF, persistent atrial fibrillation; HBP, high blood pressure; DM, diabetes mellitus; CAD, coronary artery disease; LVEF, left ventricular ejection fraction; LAD, left atrium diameter; NT-proBNP, N-terminal pro-B-type natriuretic peptide.

### Procedural characteristics in the matched population

**Figure 3:**
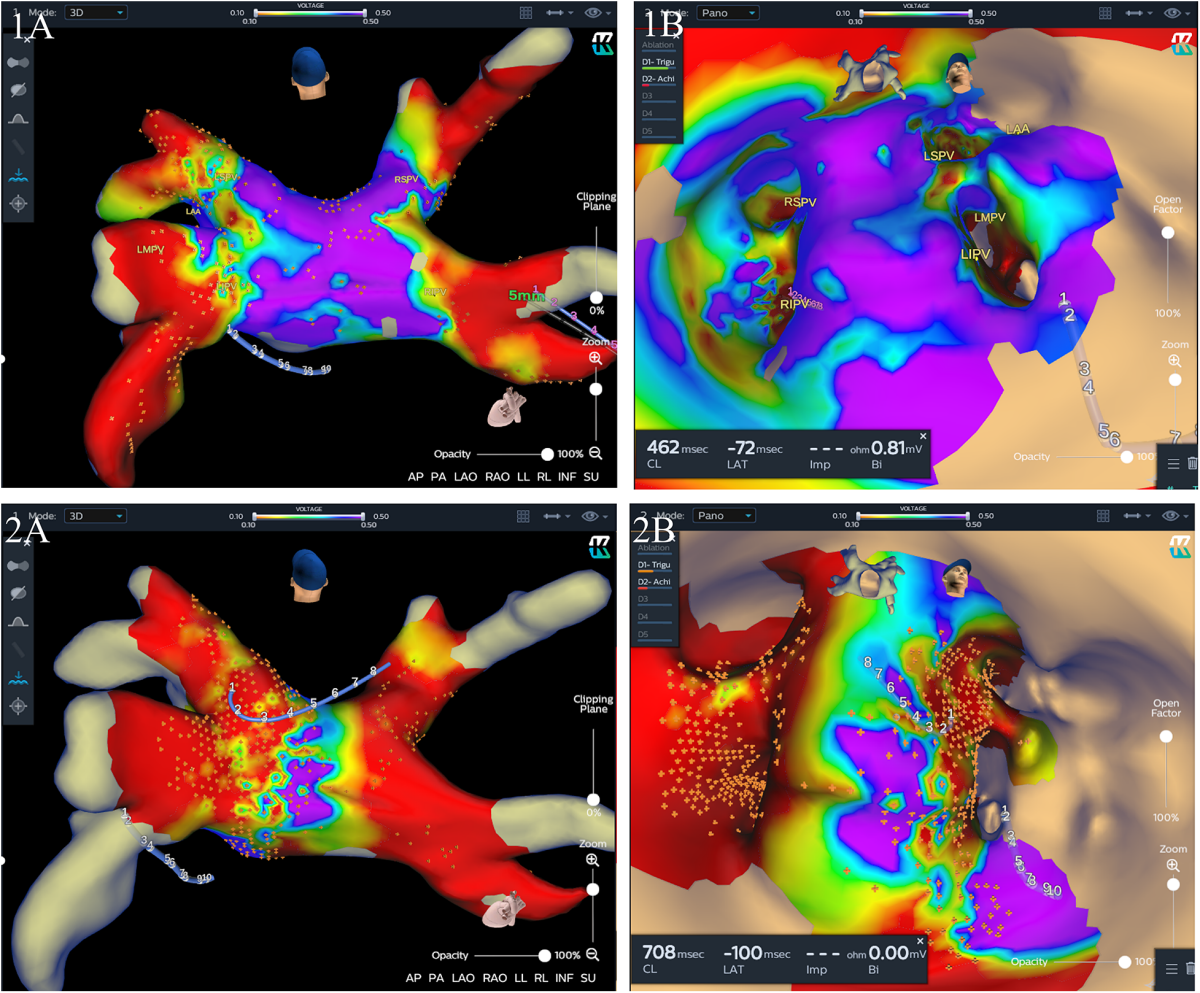
Three-dimensional (3D) electroanatomic maps (voltage cutoff: 0.1–0.5 mv) performed immediately after two freezing cycles for each PV and after additional cryoablation guided by mapping for one patient. The KODEX-EPD system offers two options for the surgeon to visualize the cardiac anatomy, a conventional shell of the heart chamber (left) and a flattened 3D panoramic view (“PANO” view) that was opened virtually across the 3D surface and offered a visualization of the endocardial surface (right). (**1A** and **1B**) A 3D image of the left atrium in a posterior-anterior (PA) view (**1A**) and “PANO” view (**1B**) illustrating residual local signals over 0.1 mv in the RSPV and left intervenous carina region immediately after two freezing cycles for each PV. (**2A** and **2B**) A 3D image of the left atrium in a PA view (**2A**) and PANO view (**2B**) illustrating a wide antral PVI including the carina region after additional cryoablation guided by mapping. LSPV, left superior pulmonary vein; LIPV, left inferior pulmonary vein; RSPV, right superior pulmonary vein; RIPV, right inferior pulmonary vein.

Procedural data before and after matching are listed in Table 2. Generally, PVI was successfully achieved in all PVs at the end of the index procedure. In the mapping group, after isolation of all four PVs, residual potential at the PV antrum was found in 30 (40.5%) patients, including 18 (24.3%) patients in the carina region, 8 (10.8%) at the roof of the LSPV antrum, 5 (6.8%) at the roof of the RSPV antrum, and 4 (5.4%) at the inferior wall of the LIPV antrum, and additional CB applications were delivered. 3D electroanatomatic maps obtained before and after additional CB applications were shown in Figure 3. While a longer fluoroscopic time and greater fluoroscopic exposure were observed in the mapping group [26.97 ± 8.07 vs. 23.76 ± 8.36, *P *= 0.023; 339 (181–586) vs. 224 (133–409), *P* = 0.012], there were no significant differences between the two groups in terms of procedural duration and LA dwell time (104.10 ± 18.76 vs. 102.45 ± 21.01, *P* = 0.619; 83.52 ± 17.01 vs. 79.59 ± 17.96, *P* =* *0.177). We defined LA dwell time as the time from the end of transseptal puncture to removal of the sheaths immediately after the end of the procedure. The total number of cryoapplications for each patient in the mapping group was similar to that in the no-mapping group (9.80 ± 1.52 vs. 9.68 ± 1.80, *P* = 0.693). Procedural complications were observed in two (2.7%) patients in the mapping group and two (2.7%) in the no-mapping group (*P* = 1). Two patients (one in each group) experienced a transient PNP and recovered before the end of the procedure. One patient in each group had groin hematoma (incidence rate 1.4%). No tamponades, no atrioesophageal fistulae, and no deaths were reported.

**Table 2:** Procedural characteristics and complications before and after matching.

Baseline characteristics	Non-matched group			Matched group		
	Mapping group (*n* = 77)	No-mapping group (*n* = 253)	*P*-value	Mapping group (*n* = 74)	No-mapping group (*n* = 74)	*P*-value
Successful PVI at the end	77 (100%)	253 (100%)	1	74 (100%)	74 (100%)	1
Total number of cryoapplications	9.91 ± 1.44	9.63 ± 1.77	0.213	9.80 ± 1.52	9.68 ± 1.80	0.693
Additional cryoablations after mapping	30 (39.0%)	–	–	30 (40.5%)	–	–
Average points per map	848 (688–1,027)	–	–	856 (680–1,061)	–	–
LA dwell time, min	84.64 ± 18.46	78.44 ± 20.44	0.018	83.52 ± 17.01	79.59 ± 17.96	0.177
Procedural duration, min	105.09 ± 19.73	101.94 ± 23.00	0.281	104.10 ± 18.76	102.45 ± 21.01	0.619
Fluoroscopic time, min	27.38 ± 8.33	23.60 ± 7.91	<0.001	26.97 ± 8.07	23.76 ± 8.36	0.023
Fluoroscopic exposure, mGy	339 (179–577)	257 (137–418)	0.027	339 (181–586)	224 (133–409)	0.012
Procedural complications	3 (3.9%)	6 (2.4%)	0.749	2 (2.7%)	2 (2.7%)	1

Values are reported as mean ± standard deviation, median, and first to third interquartile range or *n* (%). PVI, pulmonary vein isolation; LA, left atrium.

### Follow-up and clinical outcomes in the matched population

**Figure 4:**
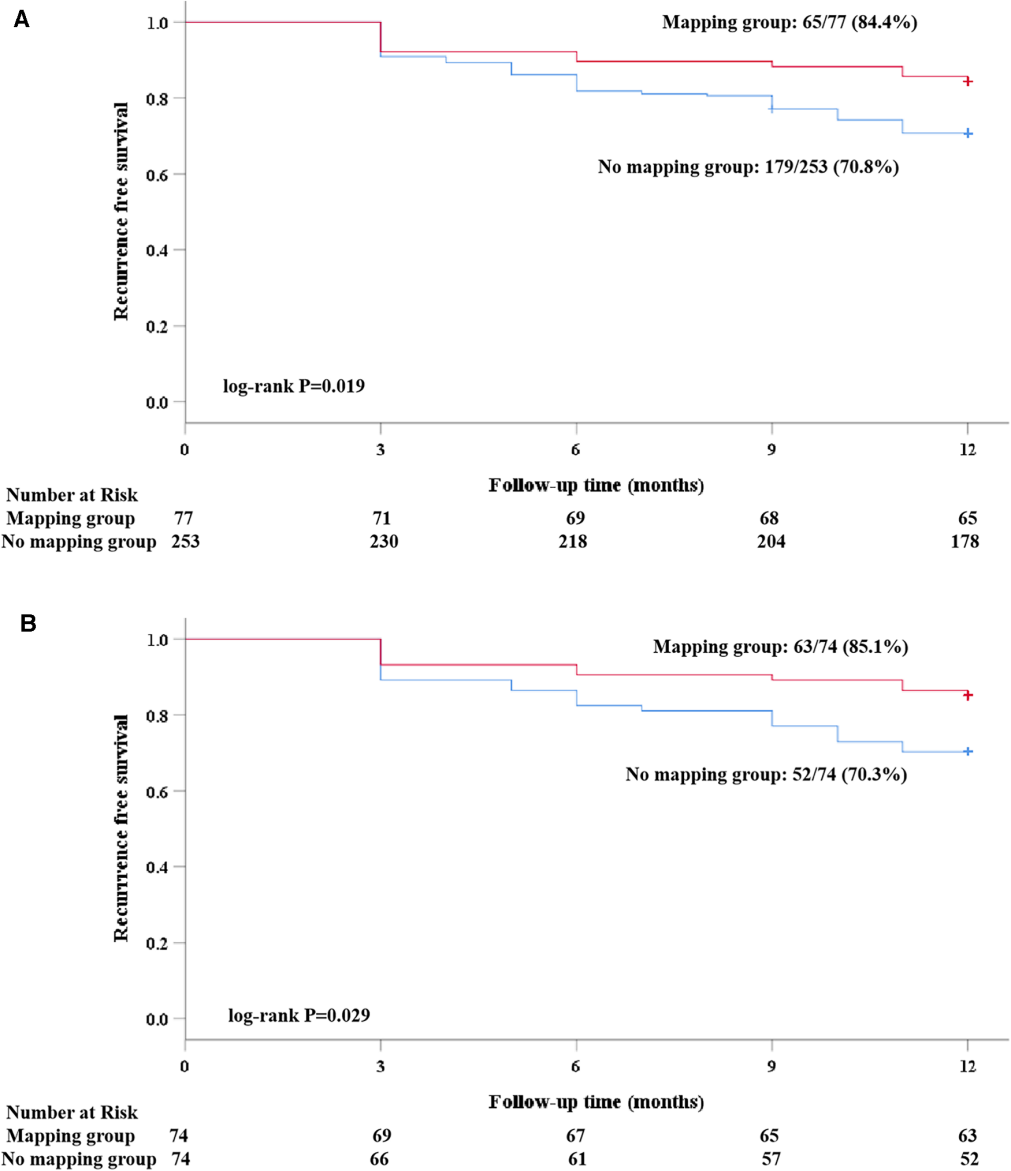
A Kaplan–Meier analysis of freedom from recurrence of AF/atrial tachyarrhythmia before and after propensity score matching. (**A**) Before matching; and (**B**) after matching).

Clinical follow-up was obtained either by the scheduled 7-day Holter in 24 (16.2%) patients or by the 24-h Holter in the remaining patients. A total of 13 (17.6%) patients in the mapping group and 11 (14.9%) in the no-mapping group received 7-day Holter monitoring (*P* = 0.656). After a follow-up of 12 months, 33 (22.3%) patients experienced clinical recurrence. The rate of clinical recurrence identified by the 7-day Holter was similar to that of the 24-h Holter (20.8% vs. 21.8%, *P* = 0.918). Among five patients who were identified as having clinical recurrence by the 7-day Holter, two (40.0%) were found to be recurrence-free at the first 24 h. The Kaplan–Meier rate of freedom from clinical recurrence at 12 months in the mapping group was significantly higher than that in the no-mapping group (before matching, 84.4% vs. 70.8%, Log-rank *P* = 0.019, Figure 4A, after matching, 85.1% vs. 70.3%, Log-rank *P* = 0.029, Figure 4B). No significant differences were found between the groups with regard to AFL/AT recurrence and early recurrence within the blanking period (Table 3). Out of 9 (12.2%) patients in the mapping group and 11 (14.9%) in the no-mapping group who experienced early recurrence, 3 patients in the mapping group and 4 in the no-mapping group underwent electrical cardioversion and the others received AADs to restore their sinus rhythm. AADs (Class I or III) were suspended for all patients at the 3-month follow-up. After the blanking period, AADs (Class I or III) were prescribed less frequently because of clinical recurrence or frequent atrial premature beats both in the mapping group and in the no-mapping group (21.6% vs. 39.2%, *P* = 0.020).

**Table 3:** Clinical outcomes before and after matching.

	Non-matched group			Matched group		
	Mapping group (*n* = 77)	No-mapping group (*n* = 253)	*P*-value	Mapping group (*n* = 74)	No-mapping group (*n* = 74)	*P*-value
Clinical recurrence	12 (15.6%)	74 (29.2%)	0.017	11 (14.9%)	22 (29.7%)	0.030
AF recurrence	11 (14.3%)	70 (27.7%)	0.017	10 (13.5%)	20 (27.0%)	0.041
AFL/AT recurrence	1 (1.3%)	5 (2.0%)	1	1 (1.4%)	2 (2.7%)	1
Early recurrence within the blanking period	9 (11.7%)	33 (13.0%)	0.847	9 (12.2%)	11 (14.9%)	0.631
AADs after the blanking period	16 (20.8%)	90 (35.6%)	0.015	16 (21.6%)	29 (39.2%)	0.020
Repeat ablation for AF	1 (1.3%)	8 (3.2%)	0.632	1 (1.4%)	3 (4.1%)	0.62
Death	0	1 (0.4%)	1	0	0	1
Stroke	0	2 (0.8%)	1	0	1 (1.4%)	1

Values are reported as *n* (%). AADs after the blanking period include the prescription of Class I or III AADs in case of AF/AT recurrence or frequent atrial premature beats.

AF, atrial fibrillation; AFL, typical atrial flutter; AT, atrial tachyarrhythmia; AADs, antiarrhythmic drugs; TIA, transient ischemic attack.

In the sensitivity analysis, the use of the high-density mapping system was associated with a significantly lower incidence of clinical recurrence [hazard ratio (HR), 0.45; 95% confidence interval (CI), 0.24–0.84; *P* = 0.012], which was generally consistent with the results obtained in the matched population [HR, 0.46; 95% confidence interval (CI), 0.23–0.96; *P* = 0.038] (Table 4).

**Table 4:** Associations between mapping system use and outcomes with propensity score matching analyses and sensitivity analyses.

Characteristics	HR	95% CI	*P*-value
Propensity score matching analysis			
Clinical recurrence	0.46	0.23–0.96	0.038
Sensitivity analysis (modeling with multivariate Cox regression)			
Clinical recurrence	0.45	0.24–0.84	0.012

Multivariate Cox regression was performed in the non-matched group (*n* = 330) and adjusted for age, sex, type of AF (paroxysmal, persistent, or long-standing persistent), body mass index, hypertension, diabetes mellitus, coronary artery disease, heart failure, history of stroke, drinking, smoking, B-type natriuretic peptide (BNP), LAD, and LVEF, which were previously used for propensity score estimation.

HR, hazard ratio; CI, confidence interval.

During the 12-month follow-up, one patient in the no-mapping group was admitted to the hospital for stroke. No other complications such as cardiac tamponade and atrial-esophageal fistula occurred in the matched population.

## Discussion

In this study, we described the use of high-density mapping in contrast to no mapping for cryoballoon PVI at our center. We found that: (1) following the conventional CB PVI, voltage mappings demonstrated that additional ablations were necessary in 30 (40.5%) patients to achieve an extensive PVI including the PV-LA junction areas and carina regions. (2) An extensive PVI identified by the mapping system was associated with a significantly lower incidence of 1-year clinical recurrence compared with conventional PVI without the mapping system (3). The use of the mapping system was associated with a longer fluoroscopic time, while no significant differences were found with regard to procedural duration and LA dwell time between the mapping and the no-mapping groups.

The CB catheter is an anatomically based ablation device that has been widely used for PVI with a favorable safety profile (5). Because of the spherical structure of the CB and size mismatch of the balloon with the PV ostium, the nature of the lesion ablated by CB-A highly depends on the PV-LA anatomy. Given that cryoablation does not involve the simultaneous isolation of the superior and inferior PV, the carina area located between ipsilateral PVs, which is the thickest segment in the PV root, may become the weak link in achieving durable PVI (19). In the conventional cryoablation procedure, the clinical judgment on PVI is based on the results of an intracardiac electrogram, and conduction gaps in the carina region may be ignored. In this context, voltage mapping with a high-density mapping system after PVI may help identify residual conduction in the carina region. In a study by Tadfumi et al. (9), residual local signals of over 0.1 mv in the carina region after CB-based PVI were found in 17 (19.8%) patients. Masateru Takigawa et al. (20) found that additional carina ablation was needed in 182 (26.1%) patients to eliminate all PV potentials (PVPs). In accordance with these published data, we also found residual carina conduction in 18 (24.3%) patients.

In a recent study by Tadfumi et al. (9), a procedure that covered an extensive isolation area that included the carina region seemed to be superior to PVI alone for achieving freedom from atrial arrhythmia by CB-A, suggesting the importance of the carina region for clinical success after cryothermal PVI. In the present study, the carina regions were targeted under the guidance of a high-resolution mapping system and patients in the mapping group were less likely to demonstrate clinical recurrence compared with those in the no-mapping group. The improved recurrence-free survival rates of carina ablation may be explained by the anatomical and electrophysiological properties of carina. On the one hand, the intervenous carina region has been extensively observed as a common trigger for AF (21, 22), and the ablation of carina may indicate an elimination of the remaining triggers in the carina regions. On the other hand, myocardial fibers in the carina region often form bridges in the epicardial site (23, 24), and only two freezing cycles at the ostial level are sometimes not sufficient for creating a complete transmural lesion. Non-transmural lesions identified by voltage mapping may help recover excitability and lead to AF recurrence in the remote period (25).

Compared with the outcomes in patients in the no-mapping group, the improved outcomes at 1 year in those in the mapping group could be attributed to additional ablation within the PV-LA junction. In the present study, residual potential within the PV-LA junction was found in 5.7% (17/296) PVs after high-density mapping. Yokoyama et al. (13) demonstrated that PV antral remapping after PVI without the use of the 3D mapping system could reveal residual PV potential in 4.3% of PVs. Because the isolation line of CB-PVI is sometimes located in the inner regions for a large PV, residual PV potential may be recorded at the proximal antrum after complete PV ostium (distal) isolation (26). In our research, the high-density mapping system provided a precise location of the PV-LA junction and the Achieve mapping catheter could record enough points for a more accurate detection of unrecognized PVPs within the PV-LA junction, which may be responsible for confirming the clinical recurrence of AF.

The use of the 3D mapping system has been proved to be associated with a shorter procedure time and fluoroscopy duration when compared with no mapping using RFA for AF (27–29). Our study focused on the use of the mapping system in CB-A, and the fluoroscopic time significantly increased, but the total procedure duration was similar to that without mapping. It was obvious that additional cryoablation to achieve an extensive PVI necessitated extra fluoroscopic time to obtain adequate balloon-tissue contact, especially when the non-occlusion technique was adopted. As for procedural duration, the benefits of 3D mapping may compensate for the additional ablation time. In our study, the total number of cryoapplications for each patient in the mapping group was similar to that in the no-mapping group. Although additional ablation was performed to achieve a wide antral isolation, the number of cryoapplications needed to achieve PVI was smaller in the mapping group than in the other group. On the one hand, the KODEX-EPD cryoablation occlusion tool helped in achieving improved PVI by indicating the degree of PV occlusion and leakage navigation, thus helping to avoid cryoablation with poor balloon-tissue contact (30, 31). On the other hand, continuous voltage mapping during cryoablation in the mapping group helped to locate low-voltage activity within the PV (32). As a result, the use of the mapping system was thought to be associated with a significantly shorter procedural time in recent studies (33, 34).

## Limitations

Several limitations should be acknowledged in the present study. First, this was a non-randomized observational study. Although propensity score matching was performed to reduce selection bias, there might exist unmeasured confounding between the treatment groups. As a result, our findings should be considered hypothetical, and randomized trials need to be performed to validate the results. Second, our results might not be applicable for longstanding persistent AF, considering the relatively small sample in which longstanding persistent AF constituted only 14.2%. Finally, the scheduled 7-day Holter was available only for 24 (16.2%) patients, and therefore, asymptomatic episodes may have occurred unnoticed, and our success rate may have been overestimated.

## Conclusions

High-density mapping after cryoablation could help identify a non-transmural lesion at the PV antrum, and wide PVI that includes the intervenous carina region, guided by high-density mapping system, resulted in a lower recurrence rate for patients with AF compared with PVI alone without mapping.

## Data Availability

The raw data supporting the conclusions of this article will be made available by the authors, without undue reservation.
